# Supplementary material for: Earliest Olduvai hominins exploited unstable environments ~ 2 million years ago
Source: Nat Commun. 2021 Jan 7;12:3. doi: 10.1038/s41467-020-20176-2 (PMC7791053; doi:10.1038/s41467-020-20176-2)
Supplement: Supplementary file 1 — Supplementary Information [file 41467_2020_20176_MOESM1_ESM.pdf]

Supplementary Information

Earliest Olduvai hominins exploited unstable environments ~ 2 million years ago

Mercader et al.

Table of contents

Supplementary Fig. 1 | Stratigraphic correlation of Bed I near Ewass Oldupa (Loc. 63)
Supplementary Fig. 2 | Lithic analysis
Supplementary Fig. 3 | Score Plots for Principal Components 1, 2, and 3
Supplementary Fig. 4 | Energy Dispersive X-Ray Fluorescence analysis of quartzite artefacts and raw material.
Supplementary Fig. 5 | Phytolith Analysis
Supplementary Fig. 6 | Stable carbon (δ13C) and oxygen (δ18O) measurements of animal teeth from Ewass Oldupa (below Tuff IA)
Supplementary Table 1 | Stone tool technological breakdown per trench at Ewass Oldupa
Supplementary Table 2 | Descriptive statistics for stone tool assemblages from selected sites
Supplementary Table 3 | Provenance, classification, and isotopic values in animal teeth from Ewass Oldupa.
Supplementary Table 4 | Number of identified faunal specimens at Ewass Oldupa
Supplementary Note 1: Faunal Taphonomy
Supplementary References

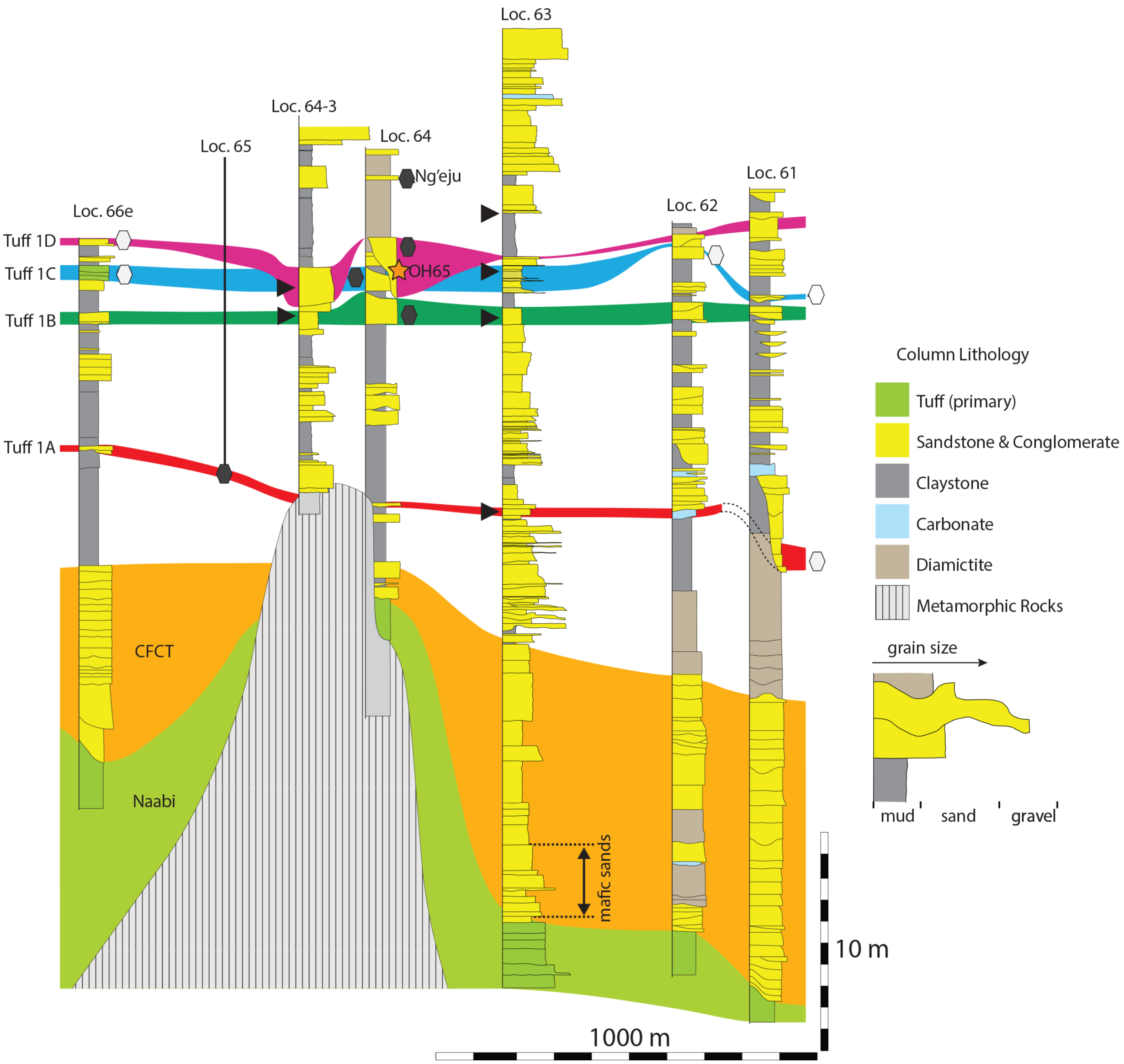

Supplementary Fig. 1 | Stratigraphic correlation of Bed I near Ewass Oldupa (Locality. 63). Sedimentary logs for Geolocality 61, 62, 64, and 66e modified from reference[1]. Sedimentary logs for Geolocality 63 and 64-3 measured, physically correlated, and geochemically fingerprinted for this study. Black triangle shows sampling location for geochemistry. Black hexagon shows sampling by reference[2-3]. White hexagon marks geochemical analysis by reference[1].

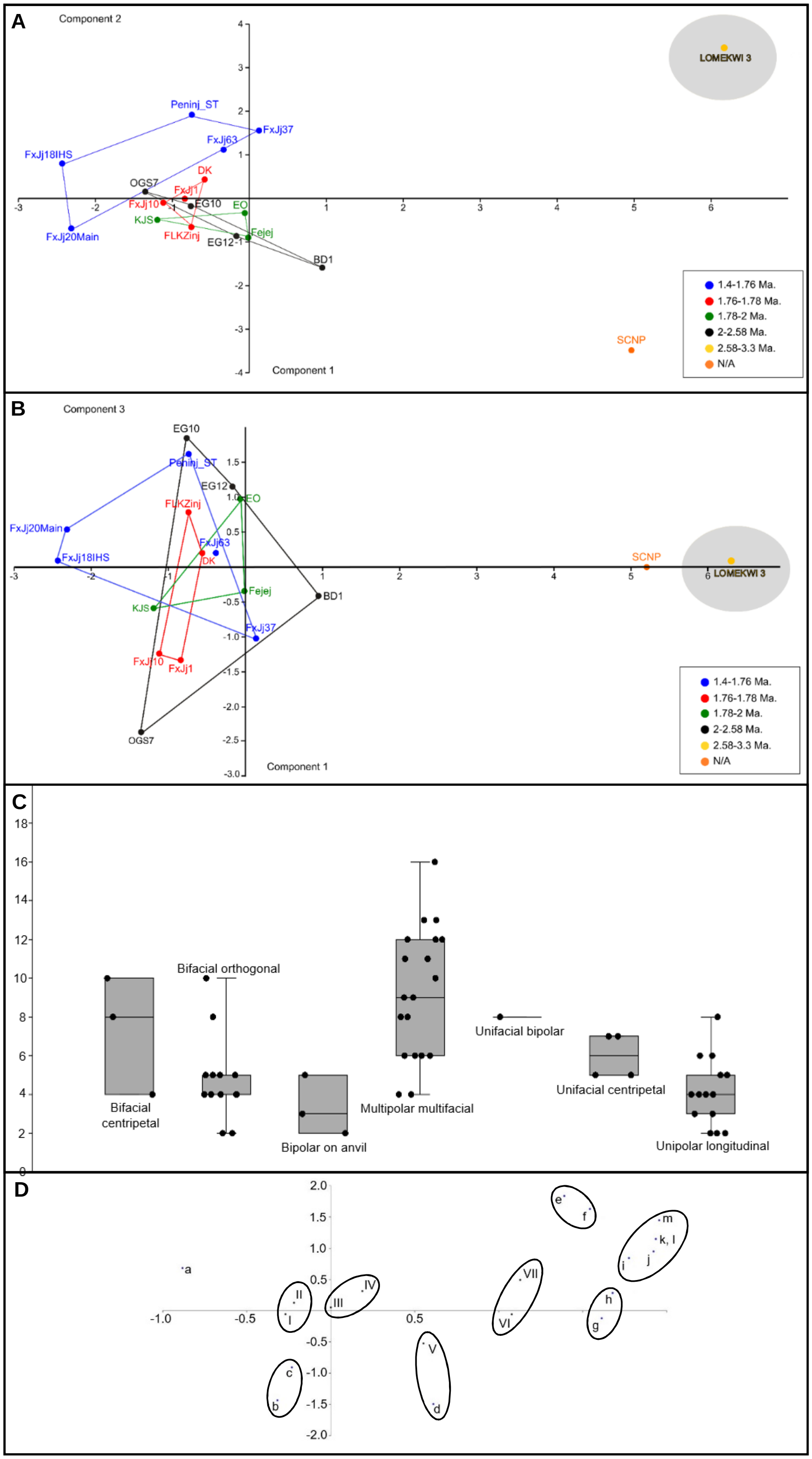

Supplementary Fig. 2 | Lithic analysis. A-B PCA of Ewass Oldupa and selected stone tool assemblages[4]. Ewass Oldupa groups with Kanjera (Kenya) and Fejej (Ethiopia) and takes on a transitional status featuring Oldowan toolkits with characteristics seen in assemblages dated to 2.58 - 2 Ma as well as those from 2.0 to 1.78 Ma. Detailed loadings are shown in Supplementary Fig. 3. See further information in Supplementary Table 2. **C** Bivariate boxplots showing median number of flake extractions from cores per knapping method. **D** Correspondence analysis establishes relationships between raw materials (Roman numerals) and stone reduction stage (letters): I. Gray quartzite subtype 1. II. White quartzite subtype 3. III. Ignimbrite. IV. Gray quartzite subtype 2. V. Gneiss. VI. Red quartzite. VII. Undetermined. (a. Flake. b. Core. c. Flake fragment. d. Broken flake. e. Chopper, Spheroid. f. Hammer. g. Core fragment. h. Core on flake i. Retouched tool. j. Hammer piece. k,l. Manuports. m. Broken hammer.)

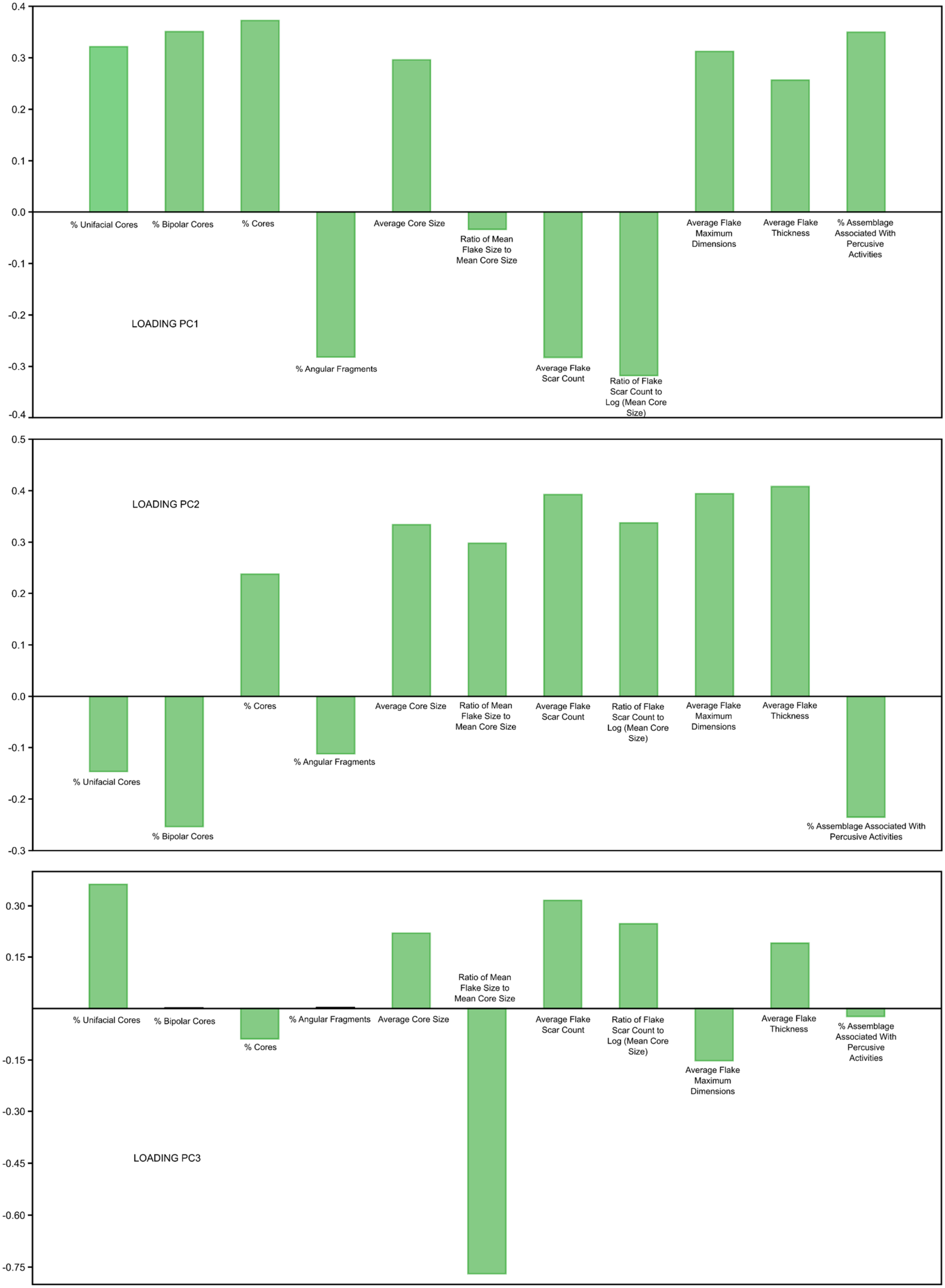
LOADING PC1
0.4
0.3
0.2
0.1
0.0
-0.1
-0.2
-0.3
-0.4
% Unifacial Cores
% Bipolar Cores
% Cores
% Angular Fragments
Average Core Size
Ratio of Mean Flake Size to Mean Core Size
Average Flake Scar Count
Ratio of Flake Scar Count to Log (Mean Core Size)
Average Flake Maximum Dimensions
Average Flake Thickness
% Assemblage Associated With Percusive Activities
LOADING PC2
0.5
0.4
0.3
0.2
0.1
0.0
-0.1
-0.2
-0.3
% Unifacial Cores
% Bipolar Cores
% Cores
% Angular Fragments
Average Core Size
Ratio of Mean Flake Size to Mean Core Size
Average Flake Scar Count
Ratio of Flake Scar Count to Log (Mean Core Size)
Average Flake Maximum Dimensions
Average Flake Thickness
% Assemblage Associated With Percusive Activities
LOADING PC3
0.30
0.15
-0.15
-0.30
-0.45
-0.60
-0.75
% Unifacial Cores
% Bipolar Cores
% Cores
% Angular Fragments
Average Core Size
Ratio of Mean Flake Size to Mean Core Size
Average Flake Scar Count
Ratio of Flake Scar Count to Log (Mean Core Size)
Average Flake Maximum Dimensions
Average Flake Thickness
% Assemblage Associated With Percusive Activities

(previous page) Supplementary Fig. 3 | Score Plots for Principal Components 1, 2, and 3 used to compare archaeological sites studied in Supplementary Fig. 2. See further information in Supplementary Table 2.

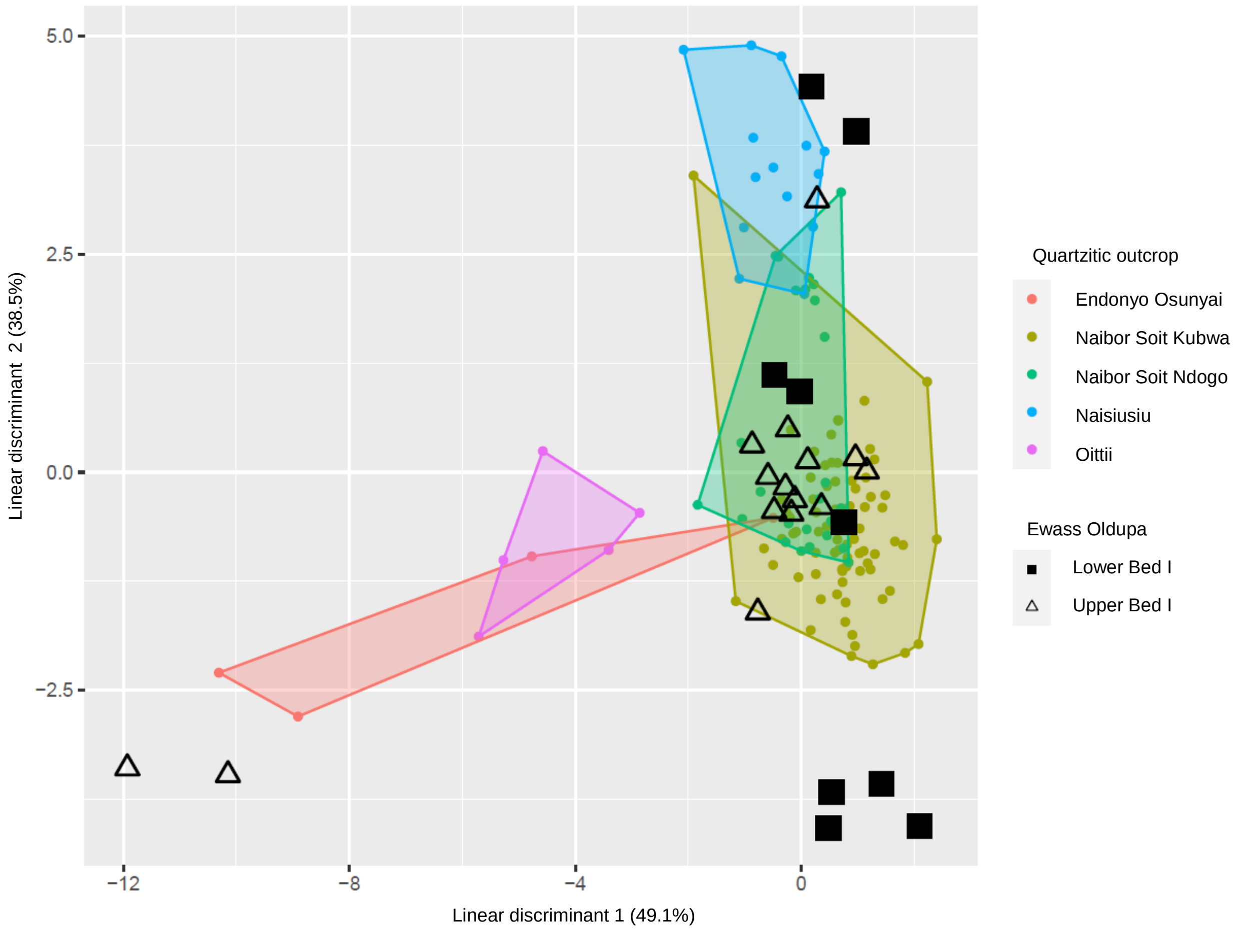

Supplementary Fig. 4 | Energy Dispersive X-Ray Fluorescence (EDXRF) analysis of quartzite artefacts and raw material. Normalised chemical concentrations of geological samples (n = 125) from five outcrops compared with artefacts (n = 24) from Ewass Oldupa. Linear Discriminant Analysis (LDA) was implemented for classification and dimensionality reduction[5]. Q-Q plots and two multivariate normality tests (Henze-Zirkler = 1.932, p = <0.001; Royston = 1108.815, p = <0.001) indicate that the chemical concentrations are non-normally distributed. Cross-validation was also performed to assess the initial hit ratio and account for unequal group sizes, which may induce over-fitting[6], as the number of predictor variables exceeded the number of observations in all but two of the sites (Naibor Soit Ndogo and Naibor Soit Kubwa).The discriminant functions of LD1 and LD2 have a trace of 49.1% and 38.5%, respectively. K_2O, Fe_2O_3, and Zn have strong positive loadings for LD1, while Al_2O_3 and MgO have moderate positive loadings for LD2. Al_2O_3, Rb, and Pb have strong negative loadings for LD1, as is the case for TiO_2 and Fe_2O_3 for LD2. Based on our LDA model, we predict sources at 75% accuracy.

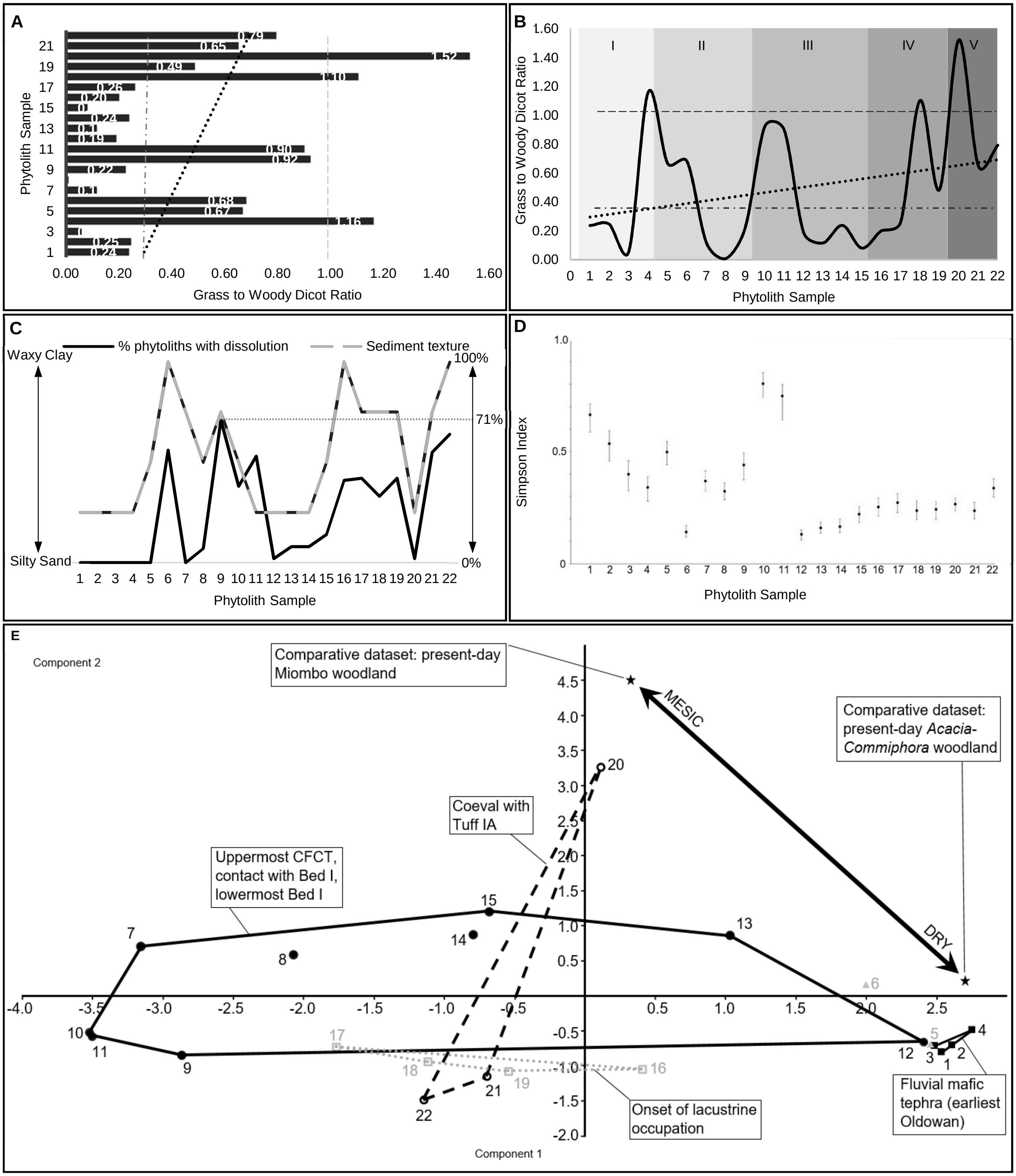

A
Phytolith Sample
Grass to Woody Dicot Ratio
0.79
0.65
1.52
0.49
1.10
0.26
0.20
0.24
0.19
0.90
0.92
0.22
0.68
0.67
1.16
0.25
0.24
B
Grass to Woody Dicot Ratio
Phytolith Sample
I
II
III
IV
V
C
% phytoliths with dissolution
Sediment texture
Waxy Clay
Silty Sand
100%
71%
0%
Phytolith Sample
D
Simpson Index
Phytolith Sample
E
Component 2
Component 1
Comparative dataset: present-day Miombo woodland
MESIC
DRY
Comparative dataset: present-day *Acacia-Commiphora* woodland
Coeval with Tuff IA
Uppermost CFCT, contact with Bed I, lowermost Bed I
Onset of lacustrine occupation
Fluvial mafic tephra (earliest Oldowan)

(previous page) Supplementary Fig. 5 | Phytolith Analysis. A The present-day comparative baseline we used to estimate grass to woody dicot ratio is from short woodland mosaics in *Acacia-Commiphora* biomes[7], where the maximum ratio is 1.00 and the average 0.30 **B** Fluctuations in grass to woody-dicot ratio throughout the column is shown in Fig. 1: Sample numbering goes from bottom to top of the sedimentary log. **C** Correlation between higher frequency in phytolith dissolution marks and fine sediments is strong ($p = 0.7078$): waxy clays contain more damaged phytoliths. The presence of dissolution marks reflects post-depositional environments leading to partial dissolution of silicon dioxide, such as matrices with high water content and alkalinity. Whether dissolution affects the ability to interpret phytoliths for a general vegetation reconstruction depends on the preservation of diagnostic morphotypes from woody dicot tissues, grasses, and other key floristic markers. **D** The Simpson index establishes dominance and evenness, thus a 0 value indicates that all morphotypes are equally represented, while a value of 1 shows dominance by one type alone. At Ewass Oldupa, Zones I and II supported extremely uneven morphotype complements, while stabilization is apparent from the middle of zone III onwards. **E** PCA of Ewass Oldupa phytolith samples (The first three components of the PCA explain 86.85% of the variance.) Each convex hull groups samples per provenance in the stratigraphic column. The modern comparative dataset comes from well-established biomes representing a continuum along which the expected palaeoenvironments would fall: from *Acacia-Commiphora* [mean distribution after bootstrapping (n=1000) phytoliths contents of 35 samples][7] to Miombo woodlands [mean distribution after bootstrapping (n=1000) the phytoliths content of n=25 samples][8-10]. The large variability detected for the upper CFCT, contact with Bed I, and lowermost Bed I suggests instability. Very few samples approach the position of the calibrated modern phytolith spectra typical of established woodland environments today, whether these are mesic or dry.

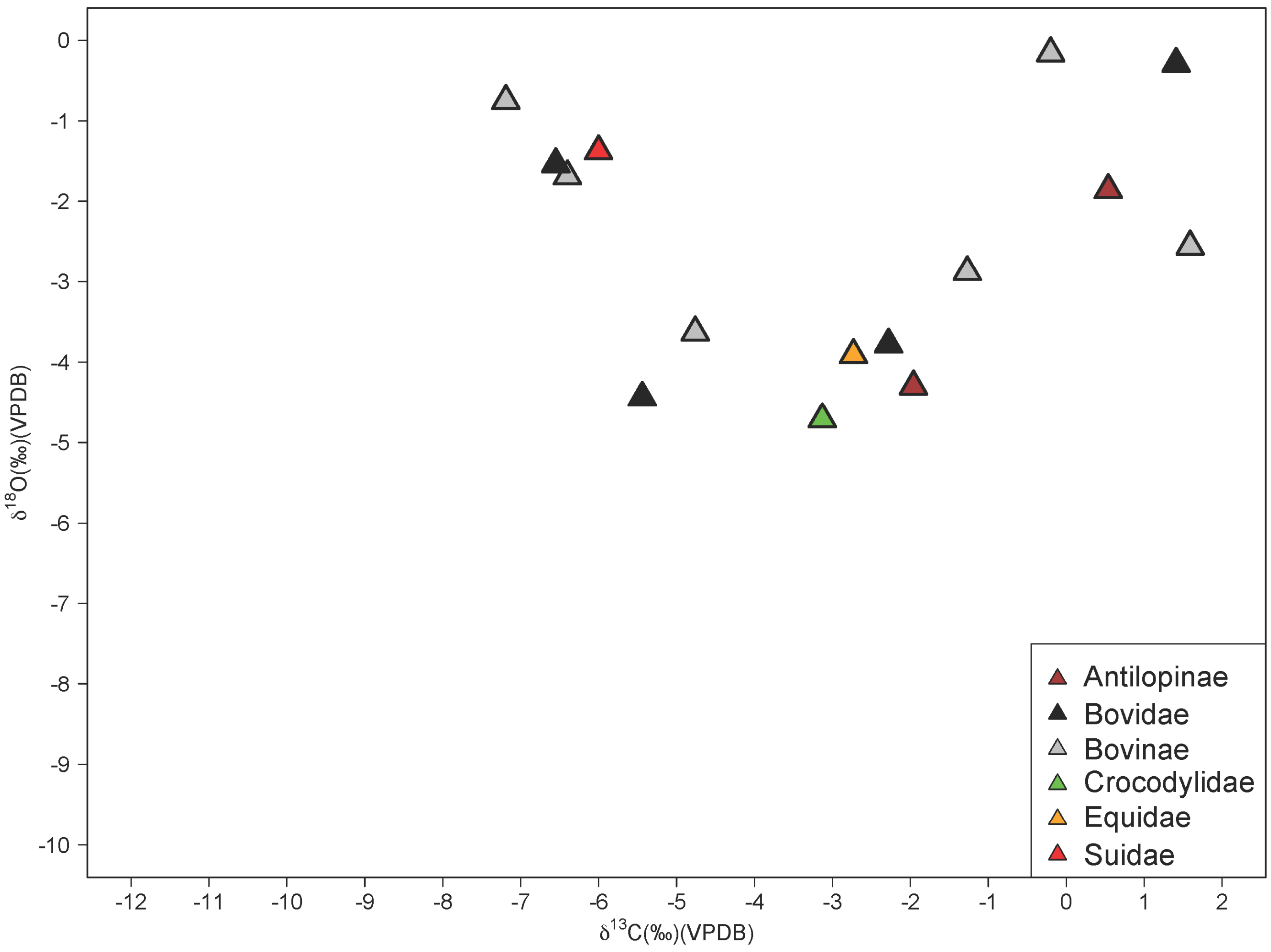

Supplementary Fig. 6 | Stable carbon (δ13C) and oxygen (δ18O) measurements of animal teeth from Ewass Oldupa (below Tuff IA). VPDB = Vienna Pee-Dee Belemnite. Enamel stable isotopes show an environmental context of open woodland, with mixed C_3 and C_4 plants, as shown by the spread of values in this graph.

Supplementary Table 1 | Stone tool technological breakdown per trench at Ewass Oldupa

		Ignimbrite	Quartzite	Basalt	Gneiss	Total
Excavation Trench T6	Cores	0	0	0	0	0
	Core Fragments	0	2	0	0	2
	Flakes	0	11	0	0	11
	Fragmented Flakes	0	1	0	0	1
	Flake Fragments	0	0	0	0	0
	Percussive Material	0	0	0	0	0
	Retouched Pieces	0	0	0	0	0
	Tools	0	0	0	0	0
	**Total**	**0**	**15**	**0**	**0**	**15**
		Ignimbrite	Quartzite	Basalt	Gneiss	Total
Excavation Trench T2	Cores	0	5	0	0	5
	Core Fragments	0	2	0	0	2
	Flakes	0	88	0	0	88
	Fragmented Flakes	0	21	0	0	21
	Flake Fragments	0	34	0	0	34
	Percussive Material	0	1	0	0	1
	Retouched Pieces	0	0	0	0	0
	Tools	0	0	0	0	0
	**Total**	**0**	**151**	**0**	**0**	**151**
		Ignimbrite	Quartzite	Basalt	Gneiss	Total
Excavation Trench T3	Cores	2	17	0	0	19
	Core Fragments	0	6	0	0	6
	Flakes	1	101	0	0	102
	Fragmented Flakes	0	16	0	0	16
	Flake Fragments	0	38	0	0	38
	Percussive Material	1	2	0	0	3
	Retouched Pieces	0	2	0	0	2
	Tools	0	2	0	0	2
	**Total**	**4**	**184**	**0**	**0**	**188**
		Ignimbrite	Quartzite	Basalt	Gneiss	Total
Excavation Trench T5	Cores	1	27	0	0	28
	Core Fragments	0	8	0	0	8
	Flakes	0	87	0	1	88
	Fragmented Flakes	0	18	0	0	18
	Flake Fragments	0	33	0	1	34
	Percussive Material	2	8	1	1	12
	Retouched Pieces	2	3	0	0	5
	Tools	0	8	0	0	8
	**Total**	**5**	**192**	**1**	**3**	**201**
		Ignimbrite	Quartzite	Basalt	Gneiss	Total
Excavation Trench T7	Cores	0	6	0	0	6
	Core Fragments	0	1	0	0	1
	Flakes	0	2	0	0	2
	Fragmented Flakes	0	1	0	0	1
	Flake Fragments	0	0	0	0	0
	Percussive Material	0	0	0	0	0
	Retouched Pieces	0	0	0	0	0
	Tools	0	0	0	0	0
	**Total**	**0**	**10**	**0**	**0**	**10**
		Ignimbrite	Quartzite	Basalt	Gneiss	Total
Excavation Trenches Combi	Cores	3	55	0	0	58
	Core Fragments	0	19	0	0	19
	Flakes	1	289	0	1	291
	Fragmented Flakes	0	57	0	0	57
	Flake Fragments	0	105	0	1	106
	Percussive Material	3	11	1	1	16
	Retouched Pieces	0	6	0	0	6
	Tools	2	10	0	0	12
	Total	9	552	1	3	565

Supplementary Table 2 | Descriptive statistics for stone tool assemblages from selected sites[4]

Site	Age (Ma.)	% Unifacial Cores	% Bipolar cores	% Cores	% Angular Fragments in Detached Pieces Assemblage	Average Core Size (mm)	Ratio of Mean Flake Size to Mean Core Size	Average Flake Scar Count	Ratio of Flake Scar Count to Log (Mean Core Size)	Average Flake Maximum Dimensions (mm)	Average Flake Thickness (mm)	% of Assemblage associated with Percussive Activities
**Peninj_ST**	1.4	33.95	0	7.1	24.19	62.29	0.54	9.36	5.22	33.4	36.1	3.1
**FxJj63**	1.5	6.94	0	6.65	37.92	108.88	0.55	7.02	3.44	60.01	10.18	0.28
**FxJj37**	1.5	26.79	0	8.86	26.66	84.56	0.78	7.13	3.7	66.01	15.21	4.37
**FxJj20Main**	1.5	31.4	0	1.96	63.71	50.5	0.52	7.14	4.19	26.01	6.46	0.14
**FxJj18IHS**	1.6	37.72	0	3.49	55.13	52.88	0.7	9.14	5.3	37.01	8.31	0.18
**DK**	1.76	37.5	1.1	16.1	40.1	67.93	0.59	7.2	3.93	40.18	11.89	3
**FLKZinj**	1.76	50.9	0	3.4	56.5	76.35	0.48	5.8	3.08	36.78	11.51	1.2
**FxJj1**	1.78	20	0	13.83	46.7	54.41	0.72	5.4	3.11	38.98	11.3	0.63
**FxJj10**	1.78	8	0	6.29	48.43	58.55	0.69	5.32	3.01	40.38	11.93	0.19
**KJS**	1.95	10.1	0	11.21	50.4	56.28	0.58	5.4	3.09	32.6	9.9	0.07
**Fejej**	1.95	59.8	0	3.52	35.95	58.3	0.63	5.1	2.89	36.9	10.7	7.01
**EO**	2	34.48	5.17	10.3	18.76	70.9	0.43	6.3	3.40	30.37	12.47	2.83
**OGS7**	2.56	14	0	3.78	44.94	44.14	0.89	5.43	3.3	39.1	11.8	1.08
**EG10**	2.56	75	0	5.61	57.25	83.33	0.43	7.25	3.77	36.1	13.6	0
**EG12**	2.56	78	0	1.5	41.32	74.45	0.46	5.38	2.87	34.6	12.8	0
**BD1**	2.58	76	0	13	23.37	58.03	0.56	2.9	1.64	32.36	9.35	1.33
**LOM3**	3.3	100	36.14	55.7	20	147.8	0.72	4.7	2.17	120	43.9	9.4
**SCNP**	0	100	100	21	28.26	71.3	0.44	1	0.54	44.34	13.2	25.2

Supplementary Table 3 | Provenance, classification, and isotopic values in animal teeth from Ewass Oldupa.
Isotope values in ‰. Size categories as per reference[59] VPDB = Vienna Pee-Dee Belemnite; SD = Standard deviation.

Trench	Number	Family/Subfamily	Tribe	Age	Tooth Sampled	Size	$\delta^{13}C$ ‰ (VPDB)	SD $\delta^{13}C$	$\delta^{18}O$ ‰ (VPDB)	SD $\delta^{18}O$
T2	F1088	Bovidae		Old Adult	RI_2	3	-5.4	0.11	-4.4	0.08
T2	F1019	Bovinae	Tragelaphini	Prime Adult	LM^3	3	1.6	0.12	-2.6	0.10
T2	F1067	Bovinae	Tragelaphini	Juvenile	Rp^2	3	-1.3	0.12	-2.9	0.07
T2	F1085	Bovinae	Tragelaphini	Juvenile	Rp^2	3	-6.4	0.16	-1.7	0.09
T2	F623	Bovinae	Tragelaphini	Juvenile	RM^2	3	-7.2	0.16	-0.7	0.13
T2	F970	Bovinae	Tragelaphini	Prime Adult	RM_3	3	-4.8	0.13	-3.6	0.07
T2	F801	Crocodylidae			M?		-3.1	0.12	-4.7	0.06
T3	F77	Antilopinae	Antilopini	Prime Adult	RM_3	2	0.5	0.13	-1.9	0.11
T3	F102	Antilopinae	Hippotragini	Prime Adult	LM_1	3	-2.0	0.11	-4.3	0.10
T3	F798	Bovidae		Prime Adult	LM_1	3	-2.3	0.11	-3.8	0.05
T3	F151	Equidae		Prime Adult	$RM^1 + RM^2$	4	-2.7	0.67	-3.9	0.09
T5	F620	Bovidae		Juvenile	LI_1	3	1.4	0.16	-0.3	0.07
T5	F612	Bovidae		Prime Adult	RM_1	3	-6.6	0.14	-1.5	0.12
T5	F339	Bovinae	Tragelaphini	Prime Adult	RM_2	3	-0.2	0.12	-0.2	0.05
T5	F617	Suidae		Prime Adult	RC_1	2	-6.0	0.13	-1.4	0.06

Supplementary Table 4 | Number of identified faunal specimens at Ewass Oldupa

Tragelaphus strepsiceros	4	1	-
Hippopotamidae cf. *Hippopotamus gorgops*	2	1?	-
Suidae cf. *Phacochoerus africanus*	5	-	1
Equus sp.	7	4	1
Panthera sp.	1	2	-
Lepus sp.	3	-	-
Crocuta crocuta	2	1	1
Erinaceus broomi	1	-	-
Theropithecus oswaldi	4	-	2
Chelonia sp.	57	9	10
Crocodylus sp.	12	1	3
Avis	13	3	1
Carnivora indet.	6	4	1
Carnivora indet. (Hyaena/Canis)	1	-	-
Perissodactyla indet.	1	-	-
Proboscidea indet.	10	-	1
Indeterminates	114	247	224
Total	436	328	278

Supplementary Note 1: Faunal Taphonomy

Trench 2 (n=762)

A total of 434 specimens preserved anatomical features and surfaces amenable to taxonomic and taphonomic analysis. The family that dominates the assemblage is the Bovidae, followed by Equidae and Suidae. Primates included *Theropithecus oswaldi*. Most bones were broken, with irregular breaks and some transverse and spiral breaks in both flat and long bones. Chi square tests show a significant difference in the breaks on long versus flat specimens (χ^2=8.6591; df= 3, p=0.034184). The dominant surface modifications are abrasion and weathering, with secondary input from crusting, trampling, etching, and staining. Anthropogenic modifications were scarce: Four specimens (size 2: three long bones, one flat bone) showed likely percussion marks while no cutmarks could be detected. Carnivore tooth marks were recorded in 3% of the bones.

Trench 3 (n=329)

The number of bones classified here is 102, with bovids dominating the assemblage in two size categories (2 and 3) to include Antilopini, Bovini, Hippotragini, and Tragelaphini. Carnivores are scarce, along with reptiles and birds. Fragmentation is very high other than teeth, phalanges, and articular bones. Surface modifications are mostly rounding, weathering, concretion, and trampling. Long fragments had transverse fractures with mixed angles indicating diagenetic fractures of dry bones. Fresh fractures were documented in two cases, displaying conchoidal notches. Carnivore tooth marks were recorded in ~7% of the specimens.

Trench 5 (n=282)

The total number of remains classified was 54. Bovids dominate (size class 3) comprising Antilopini, Bovini, and Reduncini. Other groups are scarce, *Theropithecus oswaldi*, carnivores, reptiles, and birds. Postdepositional surface modifications are mostly rounding and weathering, with limited trampling. Four specimens (~7%) showed carnivore tooth marks (long bone, scapula) and a talus of *Theropithecus oswaldi* had signs of stomach acid corrosion.

Supplementary References

1. Habermann, J. M. *et al.* Discrimination, correlation, and provenance of Bed I tephrostratigraphicmarkers, Olduvai Gorge, Tanzania, based on multivariate analyses of phenocryst compositions. *Sediment. Geol.* **339**, 115-133 (2016).
2. McHenry, L. J. A revised stratigraphic framework for Olduvai Gorge Bed I based on tuff geochemistry. *J. Hum. Evol.* **63**, 284-299 (2012).
3. McHenry, L.J. *et al.* Tuff fingerprinting and correlations between OGCP cores and outcrops for Pre-Bed I and Beds I/II at Olduvai Gorge, Tanzania. *Palaeogeogr. Palaeoclimatol. Palaeoecol.* **548**, 109630 (2020).
4. Braun, D. R. *et al.* Earliest known Oldowan artifacts at >2.58 Ma from Ledi-Geraru, Ethiopia, highlight early technological diversity. *Proc. Natl. Acad. Sci.* **116**, 11712-11717 (2019).
5. Soto, M. *et al.* Fingerprinting of quartzitic outcrops at Oldupai Gorge, Tanzania. *J. Archaeol. Sci. Rep.* **29**, 102010 (2020).
6. Soto, M. *et al.* Systematic sampling of quartzite in sourcing analysis: intra-outcrop variability at Naibor Soit, Tanzania (part I). *Archaeol. Anthropol. Sci.* **12**, 1-14 (2020).
7. Mercader, J. *et al.* Soil and plant phytoliths from the *Acacia-Commiphora* mosaics at Oldupai Gorge (Tanzania). *PeerJ* **7**, e8211 (2019).
8. Mercader, J., Bennett, T., Esselmont, C., Simpson, S. & Walde, D. Phytoliths in woody plants from the Miombo woodlands of Mozambique. *Ann. Bot.* **104**, 91-113 (2009).
9. Mercader, J. *et al.* Poaceae phytoliths from the Niassa Rift, Mozambique. *J Archaeol. Sci.* **37**, 1953-1967 (2010).
10. Mercader, J., Bennett, T., Esselmont, C., Simpson, S. & Walde, D. Soil phytoliths from miombo woodlands in Mozambique. *Quat. Res.* **75**, 138-150 (2011).
