# Supplementary material for: Earliest Olduvai hominins exploited unstable environments ~ 2 million years ago
Source: Nat Commun. 2021 Jan 7;12:3. doi: 10.1038/s41467-020-20176-2 (PMC7791053; doi:10.1038/s41467-020-20176-2)
Supplement: Supplementary file 3 — Reporting Summary [file 41467_2020_20176_MOESM3_ESM.pdf]

nature research

Corresponding author(s): Julio Mercader, Steve Hubbard, Maria Soto

Last updated by author(s): Nov 12, 2020

Reporting Summary

Nature Research wishes to improve the reproducibility of the work that we publish. This form provides structure for consistency and transparency in reporting. For further information on Nature Research policies, see our Editorial Policies and the Editorial Policy Checklist.

Statistics

For all statistical analyses, confirm that the following items are present in the figure legend, table legend, main text, or Methods section.

n/a	Confirmed	
[ ]	[x]	The exact sample size (n) for each experimental group/condition, given as a discrete number and unit of measurement
[x]	[ ]	A statement on whether measurements were taken from distinct samples or whether the same sample was measured repeatedly
[ ]	[x]	The statistical test(s) used AND whether they are one- or two-sided<br>*Only common tests should be described solely by name; describe more complex techniques in the Methods section.*
[x]	[ ]	A description of all covariates tested
[ ]	[x]	A description of any assumptions or corrections, such as tests of normality and adjustment for multiple comparisons
[ ]	[x]	A full description of the statistical parameters including central tendency (e.g. means) or other basic estimates (e.g. regression coefficient) AND variation (e.g. standard deviation) or associated estimates of uncertainty (e.g. confidence intervals)
[ ]	[x]	For null hypothesis testing, the test statistic (e.g. F, t, r) with confidence intervals, effect sizes, degrees of freedom and P value noted<br>*Give P values as exact values whenever suitable.*
[x]	[ ]	For Bayesian analysis, information on the choice of priors and Markov chain Monte Carlo settings
[x]	[ ]	For hierarchical and complex designs, identification of the appropriate level for tests and full reporting of outcomes
[x]	[ ]	Estimates of effect sizes (e.g. Cohen's d, Pearson's r), indicating how they were calculated

Our web collection on statistics for biologists contains articles on many of the points above.

Software and code

Policy information about availability of computer code

Data collection	NO SOFTWARE WAS USED
Data analysis	RStudio, ArcMap10.7, Palaeotological Statistics V.1

For manuscripts utilizing custom algorithms or software that are central to the research but not yet described in published literature, software must be made available to editors and reviewers. We strongly encourage code deposition in a community repository (e.g. GitHub). See the Nature Research guidelines for submitting code & software for further information.

Data

Policy information about availability of data

All manuscripts must include a data availability statement. This statement should provide the following information, where applicable:

- Accession codes, unique identifiers, or web links for publicly available datasets
- A list of figures that have associated raw data
- A description of any restrictions on data availability

Authors can confirm that all relevant data are included in the paper and/or its supplementary information files

Field-specific reporting

Ecological, evolutionary & environmental sciences study design

All studies must disclose on these points even when the disclosure is negative.

Study description	Multi-proxy datasets in direct association with stratified archaeological and fossil assemblages record a sustained occupation of the same place for long periods in varied geomorphic contexts and sedimentary facies. The accompanying environmental context informs Oldowan hominin ecology through indirect, classic indicators such as faunal ecology and stable isotopes from enamel (δ13C/δ18O), but also through direct evidence of vegetation physiognomy and cover, as well as regional fires, through phytolith analysis, isotopic n-alkane values, microcharcoal concentrations, and pollen spores.
Research sample	Excavated site sample, 1. Correlated with 6 other localities. Stratigraphic column > 40 m. Archaeological horizons: >7. Fossils excavated, 1373. Stone artifacts, 565. Geochemical fingerprinting of stone tools carried out on 24 specimens. Biomarker samples studied, 5. Column samples for phytolith analysis, 22. Microcharcoal/pollen samples studied, 26. Stable isotope on enamel, 15 samples. Taphonomic analysis, 1373 specimens.
Sampling strategy	The number of excavated horizons was deemed to be sufficient, as it included areal excavation of each stratigraphic member/horizon confirmed in the type geological section. The samples chosen for study represented all geological layers studied in this paper. Geochem. analysis of rocks/artifacts for provenance comprised 24 specimens from selected horizons. Samples were selected by trained geoscientists to characterize each layer, and therefore representative of all layers studied here. Palaeoecological, column samples (22-26) were more than those from any other study on phytoliths or microcharcoals in the region. Enamel work for stable isotopes was determined by the availability of dental samples optimally preserved during excavation. Same criterion applies to fossil fauna: we studied as many fossils as we retrieved, approximately 1350. Samples for X-Ray fluorescence were selected to ensure that all horizons described were included and in a number large enough to deliver significant results.
Data collection	Data were collected over the course of 2 years, since the summer season of 2018 up to March 2020, approximately. The totality of our team was present in the field at one time or another, with the exception of Tristan Carter (XRF), Siobhan Clarke/Joakim Siljedal (phytolith extraction), Patrick Roberts (Isotopes), Susan Rule/Simon Haberle (pollen/charcoal), Palmira Saladie (Taphonomy), and Alfredo Camacho/Jonathan Umbsaar (petrography/geochem). Field collection utilized digging implements consisting of screwdrivers, trowels, and dental picks. Total station: Leica.
Timing and spatial scale	Timing of sample collection is during the northern summer because of personnel availability and drier climate.
Data exclusions	none excluded
Reproducibility	Geological samples used for mineralogy (those in Table 1) relied on electron microprobe and the n measured is equal or higher than baseline to which we compared our data. Phytolith analysis relied on the tally of proxies under the microscope, and normally 3 aliquots minimum are done from the same sample to ensure consistency. Faunal analysis was done by two analysts and taxonomy was determined when both of them concurred in ID.
Randomization	Archaeological, geological, and palaeoenvironmental sampling of columns is not normally randomized, as we try to retrieve as much data as we can from the existing samples per horizon.
Blinding	We did not do studies other than geological, archaeological and ecological analysis, and investigators were blinded to group allocation during data collection and/or analysis.

Did the study involve field work? ☒ Yes ☐ No

Field work, collection and transport

Field conditions	Camp station. Daily trips to site. Return to campsite in the evening.
Location	2 57' 13" S 35 14' 55" E, 1500 m asl
Access & import/export	The Tanzania Commission for Science and Technology authorized this work under permit no. 2018-112-NA-2018-36. The Tanzanian Ministry of Natural Resources and Tourism, through its Antiquities Division, granted us permission to carry out this work (14/2017/2018) and authorities at the Ngorongoro Conservation Area allowed us to enter the protected area (BE.504/620/01/53). The export license for the materials presented in this study were obtained from the Antiquities Division (EA.150/297/01: 5/2018/2019) and the Tanzanian Executive Secretary from the Mining Commission (00001258).
Disturbance	All sites excavated were covered after completion of seasonal work.

Reporting for specific materials, systems and methods

We require information from authors about some types of materials, experimental systems and methods used in many studies. Here, indicate whether each material, system or method listed is relevant to your study. If you are not sure if a list item applies to your research, read the appropriate section before selecting a response.

Materials & experimental systems

n/a	Involved in the study
☒	☐ Antibodies
☒	☐ Eukaryotic cell lines
☐	☒ Palaeontology and archaeology
☒	☐ Animals and other organisms
☒	☐ Human research participants
☒	☐ Clinical data
☒	☐ Dual use research of concern

Methods

n/a	Involved in the study
☒	☐ ChIP-seq
☒	☐ Flow cytometry
☒	☐ MRI-based neuroimaging

Palaeontology and Archaeology

Specimen provenance	Ewass Oldupa, Oldupai Gorge, Arusha region
Specimen deposition	Upon completion of analysis and publication, all materials are to be deposited with the National Museums of Tanzania
Dating methods	We relied on published, past dating efforts that exist for the local sites and provide a solid temporal frame for our work, in addition, new chronological effors are part of future research

☐ Tick this box to confirm that the raw and calibrated dates are available in the paper or in Supplementary Information.

Ethics oversight	no ethics oversight needed

Note that full information on the approval of the study protocol must also be provided in the manuscript.
